# Electrical Characterization of Photodetectors Based on Poly(3-hexylthiophene-2,5-diyl) Layers

**DOI:** 10.3390/s140304484

**Published:** 2014-03-06

**Authors:** Juan Carlos Ferrer, José Luis Alonso, Susana Fernández de Ávila

**Affiliations:** Área de Electrónica, Universidad Miguel Hernández, Av. Universidad s/n, Ed. Quorum V, Elche 03202, Spain; E-Mails: j.l.alonso@umh.es (J.L.A.); s.fdezavila@umh.es (S.F.Á.)

**Keywords:** photodetectors, conducting polymers, P3HT, organic devices

## Abstract

This paper presents the electrical characteristics of solution-processed organic photodetectors based on poly(3-hexylthiophene-2,5-diyl) semiconducting polymer layers deposited by spin-coating on interdigitated metal electrodes. Four different electrode shapes have been used for this study in order to appraise the optimum electrode geometry. The measurement of the resistance as a function of the temperature reveals a transition from negative to positive temperature coefficient material around 80 °C for the polymer layers. Besides, slow reversible changes in the photodetectors conductivity were observed when moved from vacuum to the air and under illumination with a xenon lamp, which can be explained by the formation of charge transfer complexes with molecular oxygen and the polymer. The photogenerated current-light power ratio was found to be approximately linear in the 200 to 550 mW/cm^2^ range.

## 1. Introduction

Semiconducting polymers have been the focus of intense research over the past few decades as promising alternatives to inorganic semiconductors. These materials are suitable for the fabrication of low-cost electronic devices, since they exhibit interesting properties, such as the ease of processing, the ability to obtain large devices deposited on flexible substrates and cost reduction, especially when using deposition techniques from soluble polymers, such as spin-coating, that do not require complex systems, such as evaporation in an ultra-high vacuum. Typical polymer devices that have been extensively studied include light-emitting devices [1] and photovoltaic cells [2].

Among the many organic semiconductors available to develop polymer-based electronic devices, polythiophenes have probably been the most extensively investigated. Poly(3-hexylthiophene) (P3HT) is a solution processable semiconducting polymer well known for its interesting electrical properties: high mobility of holes ranging from 1.33 × 10^−5^ cm^2^/V and 3.30 × 10^−4^ cm^2^/V, depending on the molecular weight [3], and low width of the band gap (1.9 eV) [4]. For these reasons, P3HT has become a good candidate for developing organic solar cells [5] and organic field effect transistors [6]. However, the use of P3HT for these devices, as well as photodetection applications, is usually reported in combination with other compounds, such as phenyl-C61-butyric acid methyl ester (PCBM) [7], organic dyes [8], TiO_2_ nanoparticles [9] or carbon nanotubes [10].

Interdigitated, or interdigital, electrodes (IDE) have been selected for the polymer characterization, as they are among the most commonly used periodic electrode structures. IDE structures are commonly used for chemical sensing, nondestructive testing, as well as for surface acoustic wave devices and various microelectromechanical systems [11]. The geometry of the IDE structures consists of a finger-like periodic array of parallel in-plane electrodes used to build up the capacitance or the resistance of a layer deposited over the electrodes. IDE electrodes may be deposited on top of the polymer layer instead; however, it has been shown that the polymer-on-metal contacts exhibit better ohmic behavior, due to the reduced density of the defects [12]. Interdigitated electrodes have been also used as photodetectors in combination with inorganic compounds, such as TiO_2_ [13], or III-nitride semiconductors [14] and low-dimensional structures [15], well suited for ultraviolet light detection, and germanium [16] or PbSe [17], for infrared applications. Compared to IDE photodetectors based on inorganic semiconductors, their organic counterparts exhibit lower photogenerated currents and response times, which limit the fields of application. However, the organic materials are less expensive, and the layers are easier to process. Since P3HT is intended to be used in photovoltaic applications, a sensor based on this polymer could be used in applications where the light intensity is similar to the intensity of solar radiation, such as pyranometers for the monitorization of the incident solar radiation in photovoltaic fields. Besides, a slow response time should not be critical for this application in which changes of light intensity are very slow.

This paper presents the electrical characteristics of a series of photodetectors based on bare P3HT polymer layers, processed from solution, deposited on different metal IDE structures, without any further treatment. We have studied the dependence of the electrical characteristics on the temperature, illumination and shape of photodetectors.

## 2. Experimental Section

**Figure 1.**
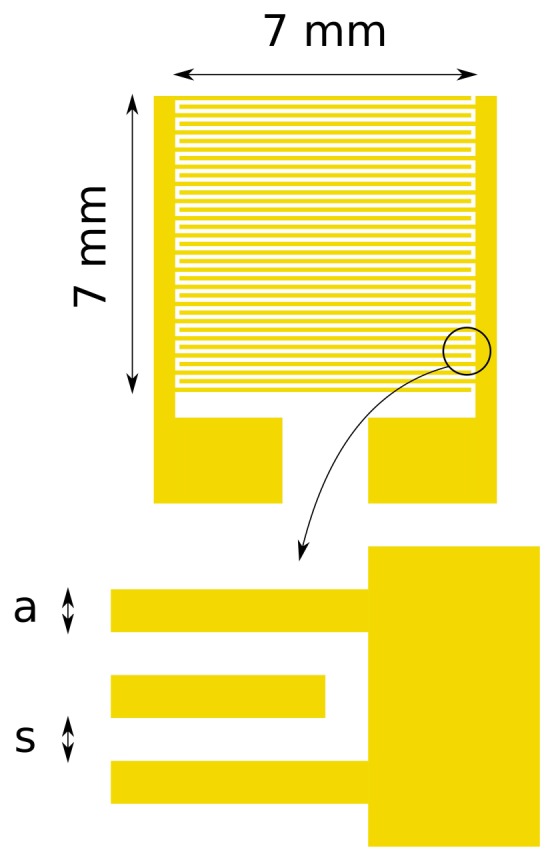
Schematics of the interdigital electrodes. Four electrodes with different finger widths (a) and separations (s) were deposited on a single substrate.

The interdigitated electrodes were produced by conventional lithographic techniques. A silicon wafer with an insulating layer of silicon oxide of 1.5 *μ*m thickness was used as the substrate. Metal layers were deposited by metal evaporation: first, a 10 nm-thick chromium layer, to improve the adherence of the upper layer, and a 100 nm gold layer on top of the chromium layer. Electrodes were patterned by chemical etching. Figure 1 shows a layout of the electrode structure. The active area in which electrode fingers extends is a square with 7 mm sides. Four different types of electrodes were produced on a single substrate, modifying the separation and width of the fingers in order to measure the electrical an optical characteristics of the polymer layer with different electrode geometries. The separation (*s*) and width (*a*) of the metal fingers are summarized in Table 1. The effective width of the serpentine gap between the two electrodes (*w*) of a single photodetector has been calculated following the work of Zaretsky *et al.* [18] and is also included in Table 1.

Chemicals were supplied by Sigma-Aldrich and used directly without further purification. Regioregular poly(3-hexylthiophene-2,5-diyl) polymer, 99.995%, was dissolved in chlorobenzene, 99.8%, since it has been reported that the use of this solvent leads to good performance of P3HT polymer photovoltaic devices [19]. For our study, a solution of P3HT in chlorobenzene (12 mg/mL) was prepared and magnetically stirred for 12 h. The solution was filtered with a filter with a pore size of 1 *μ*m, resulting in a homogeneous solution. P3HT layers were prepared by spin-coating the polymer solution at 3,000 rpm onto the substrate containing the four metal electrodes. The layers were vacuum dried in a Memmert vacuum oven at 80 °C and 25 mbar for 48 h. Electrical measurements were performed using Keithley 2400 SourceMeter equipment controlled by a LabView application via a General Purpose Interface Bus (GPIB) . A Newport solar simulator with a xenon lamp of 150 W was used as the light source.

## 3. Results and Discussion

### 3.1. Polymer Stability

A first measurement of the dark current at constant voltage was performed in order to observe the stability of the polymer when the samples were introduced from the vacuum to the air. It has been previously proven that the optical and electrical properties of polymers belonging to the family of thiophenes are sensitive to atmospheric oxygen [20]. The changes arise because the association of a molecule of low ionization potential (an electron donor) with a molecule of relatively high electron affinity (an electron acceptor) can lead to a weakly bound donor-acceptor complex or charge transfer complex. After bonding, the physical properties of the donor and acceptor are perturbed, and new electrical properties arise. This complex is loosely bound, so that its introduction is reversible. Since the samples were kept in vacuum for 48 h after spin-coating in order to dry, it is expected to find a change in electrical characteristics after the introduction of the samples into the atmosphere, due to the absorption of molecular oxygen.

**Figure 2.**
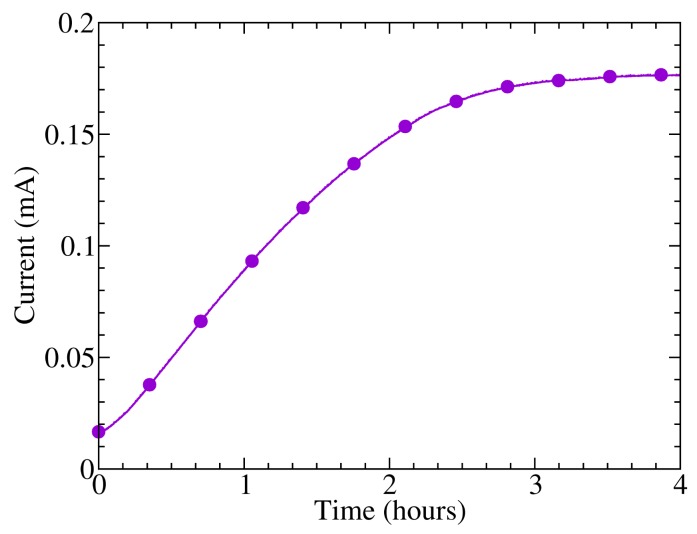
Dark current evolution of photodetector “10:20” after the introduction to the atmosphere from the vacuum.

Figure 2 shows the evolution of the current from the photodetector 10:20 biased with a voltage of 10 V in darkness immediately after its introduction into the atmosphere. It can be observed that there is an increase of the dark current during the first three hours of environmental exposure and subsequent stabilization, which could be attributed to this effect. Smaller, long-term current variations were also observed, as the sample was kept in the same conditions, probably due to the change of other environmental variables, such as temperature, as will be shown later, or humidity [21]. Besides, a recovery of the initial current values was observed as the samples were reintroduced in vacuum, producing the desorption of the oxygen dissolved in the polymer layers and a reduction in the charge donor-acceptor centers. Other authors report time responses of 40 min in thin film P3HT transistors after the application of 10 atm O_2_ from vacuum [20]. However, the change of conductivity after exposure to oxygen can take several days to reach equilibrium in the case of oligothiophenes [22], which are known to show a higher degree of crystallinity and, hence, a lower oxygen diffusion coefficient.

### 3.2. Shape of Electrodes

**Figure 3.**
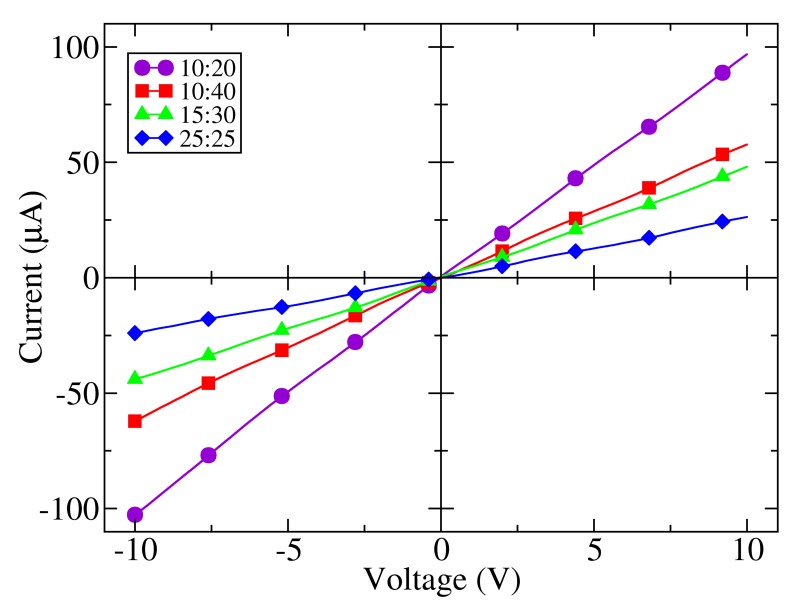
Dark current-voltage characteristics of all photodetectors at room temperature showing linear responses.

A series of measurements of the voltage-current characteristic of each of the samples at room temperature and darkness were performed to assess the dependence of the resistance of the electrodes on their geometry. As shown in Figure 3, a pure resistive behavior is found, since the polymer layer has a linear current-voltage characteristic in the range of measurement. This figure shows that the current for a particular bias is higher for shorter finger separations and wider electrode gaps, as expected for any resistive element [23]. The influence of the finger separation and electrode effective width can be observed through the comparison of samples 10:40, 10:20 and 25:25 the highest current, or minimal resistance, is found in sample 10:20 (*R*_10:20_ = 97 kΩ), with a minimum electrode spacing and a maximum width of the gap. Reducing the channel width keeping the finger separation constant increases the resistance (sample 10:40, *R*_10:40_ = 227 kΩ). A broadening of the finger separation holding the electrode gap is followed by a further increment of the resistance (sample 25:25, *R*_25:25_ = 415 kΩ). Sample 10:40 and 10:20 have the same finger separation; however, the later has a gap 1.7 times wider than the former. Sample 25:25 has almost the same effective width as sample 10:40, but a finger separation 2.5 times larger.

### 3.3. Influence of Temperature

**Figure 4.**
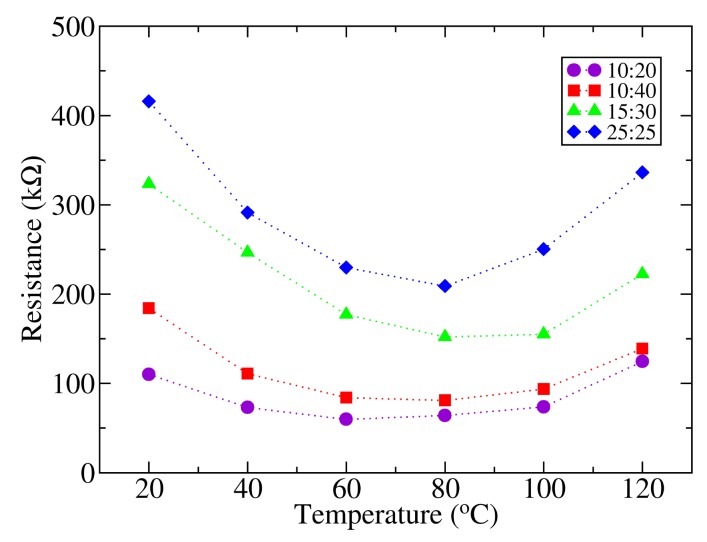
Resistance evolution of photodetectors as a function of temperature. The dotted lines are guides for the eye.

The current voltage characteristics of the samples were measured in a range of temperatures between 22 and 120 °C. The evolution of the photodetector resistance with temperature calculated from these measurements is shown in Figure 4. For low temperatures, it can be found that there is an initial decrease in resistance when the temperature is raised, reaching a minimum around 60–80 °C. From this point, the resistance increases as the temperature is further augmented. The charge transport mechanism in conjugated polymers has to be considered in order to explain this behavior. In general, the conductivity of conjugated polymers can be assumed to be the contribution of three components: tunneling transport, intra-chain transport and inter-chain transport [24]. Charge transport in P3HT is based on phonon assisted hopping of thermally activated jumps between localized states for temperatures above 40 K, (inter-chain transport), which results in an increased conductivity as the temperature is raised [25], as far as the separation between polymer *π* chains remains unaltered. However, an enlargement of the inter-chain distance as the temperature increases could result in a transition of the material from a negative to a positive temperature coefficient. Such a transition has been observed previously in fractionated P3HT layers [26]. In this paper, the transition is attributed to a reduction of the stacking of *π* polymer chains as the temperature exceeds the threshold temperature of 50 °C, which is close to the transition temperature found in the present work. Since the charge transport is achieved by a mechanism of charge hopping between *π* bonds, a separation of these results in an increased resistivity. A further reduction of the conductivity as the temperature approaches the polymer melting point is reported in the literature [27].

### 3.4. Photogenerated Current

**Figure 5.**
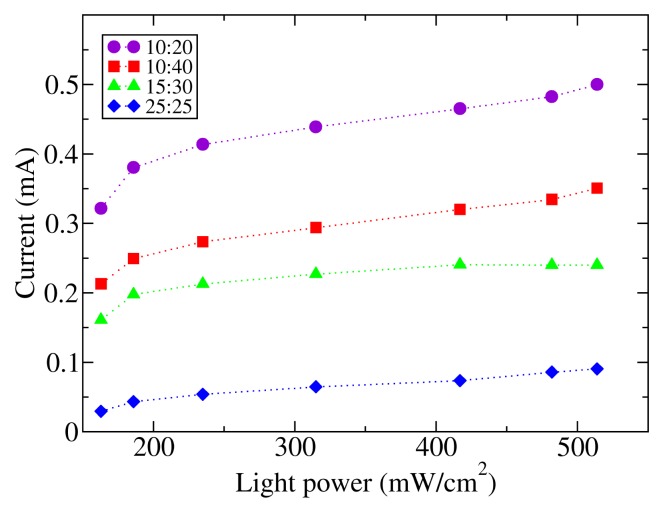
Current vs. the light power characteristics of photodetectors. The dotted lines are guides for the eye.

A series of measurements of the current with a 10 V fixed bias voltage were performed by illuminating the sample by means of the xenon lamp of a solar simulator with a power light on the polymer surface between 160 and 515 mW/cm^2^. The results show a linear dependence of the photogenerated current in relation to the incident power in the range between 200 and 515 mW/cm^2^ (Figure 5). Other studies with lower intensities show that the range of linearity extends to the dark condition [23].

**Figure 6.**
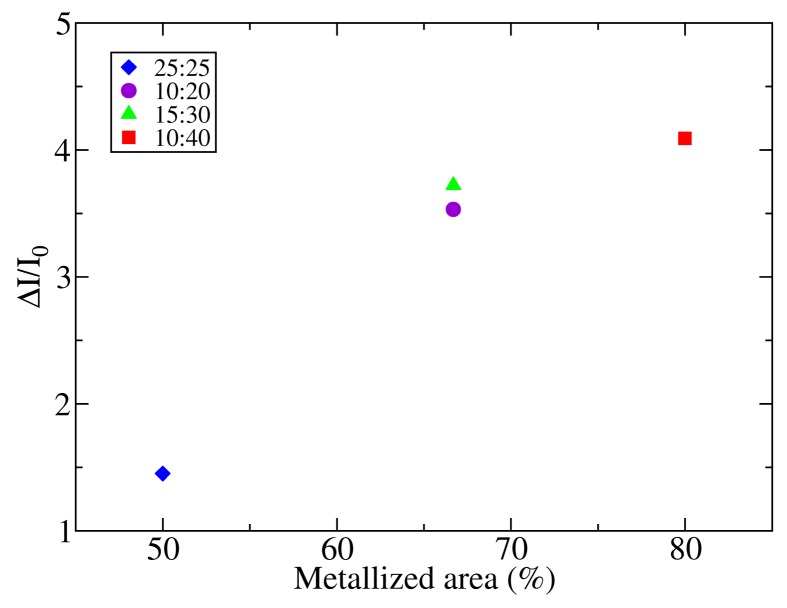
The photocurrent (Δ*I*_0_) dark current (*I*_0_) ratio *vs.* the fraction of the metal area of the photodetectors.

In order to assess the influence of the electrodes on the photodetector response, the photogenerated current relative to the dark current (Δ*I*/*I*_0_ = (*I* − *I*_0_)/*I*_0_) has been compared to the area fraction covered by the metal fingers in the four electrodes. The results have been plotted in Figure 6. The lower relative increment of current when the photodetector is illuminated is found for sample 25:25, which is the device with a lower amount of metalized area (50%). Samples 10:20 and 15:30, which have the same fraction of metalized area (67%), show similar levels of relative photogenerated current, although the absolute current of sample 10:20 is almost twice the absolute current of sample 15:30. Moreover, these samples show higher photogenerated current than sample 25:25. Finally, sample 10:40, with 80% of the active area covered with metal, shows the higher Δ*I*/*I*_0_ ratio. Hence, although the absolute current depends mainly on the finger separation (s) and gap width (w), as has been shown in Section 3.1, the key parameter that governs the sensitivity of the photodetectors is the metalized area.

In order to explain that the photogenerated current depends on the metalized area for sensors with identical total area, a comparison between the diffusion length of charge carriers in P3HT and the metal finger-to-finger separation has been considered. Diffusion lengths in P3HT range between 2.6 and 8.5 nm (see [28] and the references therein), which are values considerably low compared to the diffusion lengths of typical inorganic semiconductors. Since the separation between electrodes, which ranges between 10 and 25 *μ*m, is three orders of magnitude higher than the diffusion length of the exciton, only the charge generated near the surface of the metal can reach the contacts and contribute to the photogenerated current [29]. Thus, photodetectors with larger metalized areas would be more efficient in collecting the photogenerated charge. Besides increasing the metalized area, it has been recently demonstrated that an alternative method to increase the sensitivity of P3HT light sensors is the doping of the polymer with gold nanoparticles [30].

### 3.5. Dynamic Characteristics

**Figure 7.**
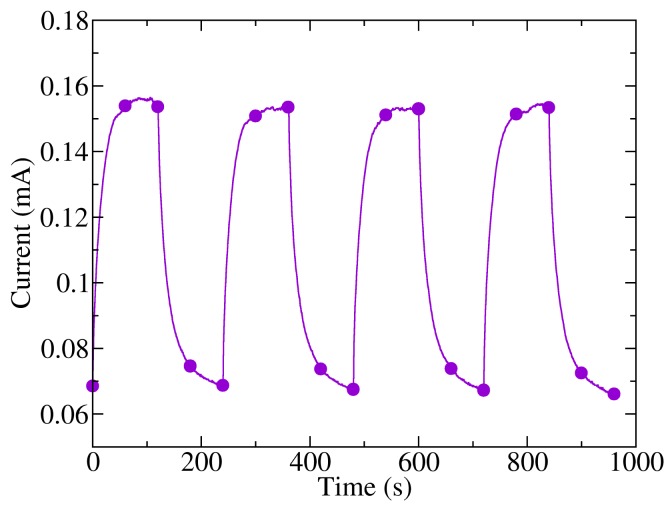
Photodetector “10:20” response to light/darkness cycles.

Finally, a study of the response of the photodetectors to changes in lighting was performed. The samples were biased with a voltage of 10 V and were exposed to pulsed dark/light cycles, alternating from darkness to 240 mW/cm^2^ light power, with a period of four minutes (two minutes with light and two minutes in darkness). The graph of the measured current in these conditions is shown in Figure 7. It can be observed that there is a slow response of the photogenerated current when abrupt changes of lighting are applied, which is in good agreement with previous results found in the literature [23]. Likewise, the rise time is slightly shorter than the fall time. Specifically, the mean time needed to reach 90% of the maximum current value was 35 s, while the fall time to 10% of the maximum current was 48 s, similar values to those found for pristine P3HT thin-film photoconductors [23] and phototransistors [31]. Whether the change of current is related to an interband transition or to a photoassisted physical or chemical process remains unclear. Although photodetectors held the maximum and minimum current levels in light and dark conditions throughout the experiment, which suggest an interband charge generation, the current transient is remarkably slow. Simulation of the response to illumination in phototransistors shows that the electric field and the carrier gradient are low. Thus, longer times should be required to relax the exceeding carrier once the light is turned off. It has been shown previously that light enhances the reversible generation of charge transfer complexes in this polymer [32], P3HT phototransistors [33,34] and P3HT solar cells [35], which could explain the slow variations of the photogenerated current. Nevertheless, it has also been found that the dark current levels after several cycles of illumination and darkness are higher than the dark current before illumination [34], suggesting that photoassisted molecular oxygen doping is not a completely reversible process.

## 4. Conclusions

We performed a study of the electrical characteristics of P3HT conducting polymer layers deposited on interdigitated electrodes by spin-coating. It has been found that reversible changes in conductivity by exposing the layers to the environment occur, probably due to absorption/desorption of molecular oxygen, which acts as a donor-acceptor center. It has been observed that there is a transition of the temperature coefficient from negative to positive, attributed to a reduction in the stacking of the *π* bonds of the polymer. The photocurrent/dark current ratio has been found to depend on the amount of metalized area, or electrode area, instead of the total photodetector active area. Finally, slow photocurrent changes have been observed as conditions change between light and darkness. Although sensitivity time responses are low, low-cost and large area applications of solution-processable P3HT-based photodetectors are feasible.

## Figures and Tables

**Table 1.** Finger separation (s), finger width (a) and effective width of the serpentine gap between electrodes (w) of the four photodetectors.

**Sample**	s (*μ*m)	a (*μ*m)	w (mm)
10:20	10	20	1,624
10:40	10	40	976
15:30	15	30	1,078
25:25	25	25	970
